# Analysis of the Plant *bos1* Mutant Highlights Necrosis as an Efficient Defence Mechanism during *D. dadantii/Arabidospis thaliana* Interaction

**DOI:** 10.1371/journal.pone.0018991

**Published:** 2011-04-21

**Authors:** Yvan Kraepiel, Jacques Pédron, Oriane Patrit, Elizabeth Simond-Côte, Victor Hermand, Frédérique Van Gijsegem

**Affiliations:** 1 INRA, UMR217, Interactions Plantes-Pathogènes, Paris, France; 2 UPMC Univ Paris 06, UMR217, Interactions Plantes-Pathogènes, Paris, France; 3 AgroParisTech, UMR217, Interactions Plantes-Pathogènes, Paris, France; Indian Institute of Science, India

## Abstract

*Dickeya dadantii* is a broad host range phytopathogenic bacterium provoking soft rot disease on many plants including *Arabidopsis*. We showed that, after *D. dadantii* infection, the expression of the *Arabidopsis BOS1* gene was specifically induced by the production of the bacterial PelB/C pectinases able to degrade pectin. This prompted us to analyze the interaction between the *bos1* mutant and *D. dadantii*. The phenotype of the infected *bos1* mutant is complex. Indeed, maceration symptoms occurred more rapidly in the *bos1* mutant than in the wild type parent but at a later stage of infection, a necrosis developed around the inoculation site that provoked a halt in the progression of the maceration. This necrosis became systemic and spread throughout the whole plant, a phenotype reminiscent of that observed in some lesion mimic mutants. In accordance with the progression of maceration symptoms, bacterial population began to grow more rapidly in the *bos1* mutant than in the wild type plant but, when necrosis appeared in the *bos1* mutant, a reduction in bacterial population was observed. From the plant side, this complex interaction between *D. dadantii* and its host includes an early plant defence response that comprises reactive oxygen species (ROS) production accompanied by the reinforcement of the plant cell wall by protein cross-linking. At later timepoints, another plant defence is raised by the death of the plant cells surrounding the inoculation site. This plant cell death appears to constitute an efficient defence mechanism induced by *D. dadantii* during *Arabidopsis* infection.

## Introduction

*Dickeya dadantii*, a broad host-range phytopathogenic enterobacterium, is the causal agent of soft rot disease on many crops, ornamentals and on the model plant *Arabidopsis thaliana* [1], [2]. *D. dadantii* virulence relies mainly on the production and secretion of plant cell wall degrading enzymes into the extracellular spaces of infected tissues [3], [4]. These include pectinases and a cellulase both secreted by a type II Out secretion system and proteases secreted by a type I Prt secretion system [5], [6]. *Out* mutants are unable to cause maceration symptoms [7] and mutants affected in the Prt secretion system are delayed in symptom progression [8]. The synthesis of the degrading enzymes is finely tuned *in vitro* by metabolic stimuli and environmental conditions and accordingly, a set of transcriptional regulators involved in cell wall degrading enzyme production have been characterized [9], [10]. This fine tuning of the production of virulence factors has also been revealed *in planta*, leading to the coordinated production of several of these factors when bacterial population has reached a certain threshold [11]. Like many other Gram-negative pathogenic bacteria, *D. dadantii* also possesses a type III Hrp secretion system, but this system has been shown to play only a minor role in pathogenesis: *hrp* mutants are less efficient in the initiation of maceration in conditions that are unfavourable to bacterial infection such as low density inocula [12], [13] or infection of semi-tolerant *Saintpaulia* plants [14]. Often, after invading its host plant, *D. dadantii* cells reside latently in the plant intercellular spaces without provoking any symptoms. In this case, disease occurs only when the environmental conditions are favourable for both massive bacterial multiplication and production of virulence factors [15], [16].

Plant defence responses against soft rot *Erwiniae* were mainly studied using *E. carotovora* (renamed *Pectobacterium*) on different host plants. In tobacco, both *Pectobacterium* and bacterial cell-free culture filtrates containing secreted plant cell wall degrading enzymes were shown to induce plant defence responses in a salicylic acid (SA)-independent manner although SA is able to induce plant resistance to this pathogen [17], [18]. In *Arabidopsis*, *Pectobacterium*_SCC1_ was shown to activate both SA- and jasmonate (JA)/ethylene-dependent plant defence signalling and the integration of these SA- and JA-signalling events involved the WRKY70 transcription factor [19], [20]. While the arsenal and modes of action of virulence factors are well characterized for *D. dadantii*, the deciphering of the plant partner's role in the interaction is still in its infancy. No monogenic resistance to *D. dadantii* has been characterized but differences in symptom severity have been reported for several crops [14], [21]. The mechanisms underlying the basal resistance against this pathogen are still largely unknown. One of the best studied processes during the interaction is competition for iron within the plant. Indeed, *D. dadantii* produces two siderophores that provide iron to the bacterium. Furthermore a link between the iron status and plant basal immunity in the *D. dadantii*/*Arabidospsis* interaction has been revealed [2], [22]–[24]. Other plant defence mechanisms are activated during *D. dadantii* infection. In parsley, the defence-related *ELI* genes were activated during the infection by wild type *D. dadantii* or different bacterial mutants, without correlation between this induction and symptom severity [25]. Recently, the importance of the abscisic acid status on soft rot maceration symptoms during infection of tomato has also been highlighted [26]. Finally, Fagard *et al.* [27] analyzed defence responses of the plant model *Arabidopsis* after *D. dadantii* infection. Hereby, infection was accompanied by an early ROS production peaking 24 hours post infection, achieved by the action of the NADPH-oxidases, mainly AtrbohD and accessorily AtrbohF, as well as by activation of marker genes of the SA, JA and ethylene signalling pathways involved in the plant immune network. ROS can directly reinforce passive barriers against pathogens - for example by chemically modifying plant cell walls - but are also important signal molecules mediating gene activation [28], [29]. *D. dadantii* is able to cope with this oxidative stress encountered *in planta* by accumulating anti-oxidant molecules like indigoïdine [30] and by producing several factors involved in cellular repair of ROS damages [31], [32]. Plant ROS production is nevertheless partly effective in counteracting disease progression since an *AtrbohD-AtrbohF* double mutant is more susceptible to the bacterial infection [27]. Analysis of the susceptibility to *D. dadantii* infection of plant mutants altered in SA and JA/ethylene signalling pathways (*sid2*, *jar1* and *coi-1* respectively) revealed that SA is not involved in resistance to *D. dadantii* while *jar1* and *coi* mutants exhibit slightly more severe symptoms, pointing to a weak involvement of JA and ethylene pathways in basal resistance [27].

These data suggest that the *D. dadantii*/*A. thaliana* interaction is a complex process and that *D. dadantii* is able to counteract all plant responses already studied. In order to identify new plant factors important for soft rot disease development, we adopted a candidate gene approach. Among a panel of *Arabidopsis* mutants already identified as altered in response to necrotrophic/macerogenic pathogens, we focused on the *bos1* mutant (for *bo*trytis *s*usceptibility), previously shown to be hyper susceptible to the two necrotrophic fungi *Botrytis cinerea* and *Alternaria brassissicola* [33], [34]. This mutant is affected in the production of the MYB108 transcription factor. Here we report the specific induction of the *Arabidopsis BOS1* gene during *D. dadantii* infection. This induction is associated with the secretion of specific bacterial pectinases. Phenotype analysis of the *bos1* mutant revealed distinct early and late responses to *D. dadantii* infection. Moreover, this work shows that a necrosis is an efficient plant defence response during this interaction.

## Results

### *D. dadantii* induces *BOS1* expression through the secretion of specific pectinases

**Figure 1:**
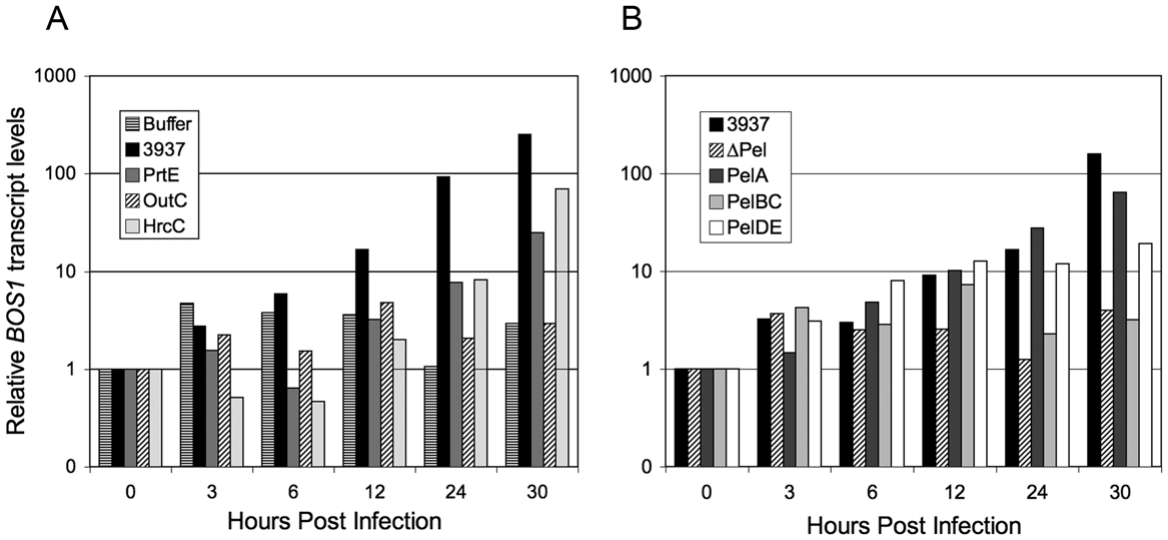
*D. dadantii* induces *BOS1* gene expression through the secretion of specific proteins. Six-week-old Col-0 wild type plants were inoculated by immersion into phosphate buffer or into 5.10^7^ cfu/mL bacterial suspensions. Rosettes were harvested at the time points indicated at the bottom and *BOS1* gene expression was analyzed by quantitative real-time RT-PCR using the *BETA-6 TUBULIN* as constitutive standard gene. **A:** analysis of the involvement of bacterial protein secretion systems in the induction of *BOS1* expression. Tested bacterial strains are 3937 wild type strain, *prtE* type I secretion system mutant, *outC* type II secretion system mutant and *hrcC* type III secretion system mutant. Relative *BOS1* transcript levels were expressed according to the reference condition (0 hour post infection) set to 1 for each genotype. This result is a representative example out of three biological replicates. **B:** analysis of the involvement of the major pectinases secreted through the type II secretion system in the induction of *BOS1* expression. Tested bacterial strains are 3937 wild type strain, *Δpel* mutant strain deficient for the production of the five major pectinases PelA to PelE, *pelA* mutant strain deficient for the production of the PelA pectinase, *pelBC* mutant strain deficient for the production of the PelB and PelC pectinases, *pelDE*, mutant strain deficient for the production of the PelD and PelE pectinases. Relative *BOS1* transcript levels were expressed according to the reference condition (0 hour post infection) set to 1 for each genotype. This result is a representative example out of two biological replicates.

The *BOS1* gene was reported to be activated by *Botrytis cinerea* [33], a necrotrophic fungus that, like *Dickeya*, provokes maceration of plant tissue by producing plant cell wall degrading enzymes. This prompted us to analyze *BOS1* transcripts accumulation during *D. dadantii* infection. Plant infection was performed by immersing whole plants in a bacterial suspension to use a non invasive mode of inoculation and to maximize the number of leaves responding to the pathogen. *BOS1* transcripts levels were followed during the first 30 h of infection and we detected *BOS1* transcripts accumulation from 12 hpi in Col-0 plants infected with the wild type bacterial strain 3937 (Fig. 1A).

In bacteria-plant interactions, signalling is very often achieved via the secretion/translocation of effector proteins. To tackle the bacterial inducing factor, we analyzed *BOS1* gene activation after infection with mutants impaired in the different *D. dadantii* secretion systems characterised. Three mutants were analyzed, *prtE*, *outC* and *hrcC* impaired in type I, II and III secretion systems respectively (Fig. 1A). No significant difference of *BOS1* transcript levels between the buffer and bacterial inoculations was observed before 24 hours post infection. From 24 to 30 hours, transcript accumulation progressively increased for wild type, p*rtE* and *hrcC* mutants. In contrast, *BOS1* transcript accumulation for o*utC* mutant remained at similar levels as after buffer inoculation. Since the type II secretion system (T2SS) encoded by *out* genes is involved mainly in the secretion of the cell wall degrading enzymes responsible for the maceration symptom, mutants affected in the production of the five major *D. dadantii* pectinases were tested for their inductive effect on *BOS1* (Fig. 1B). Because PelB and C on one hand and PelD and E on the other hand are highly similar in their amino acid sequence as well as in their enzymatic activity characteristics [35], [36], we tested the *ΔpelABCDE* mutant, deleted for the five genes encoding the major pectate lyases, the single mutant *pelA* and the double mutants *pelBC* and *pelDE*. *BOS1* expression remained low after inoculation with the *ΔpelABCDE* and the *pelBC* mutants throughout the infection while a significant level increase was observed at 24 and 30 hours post infection after inoculation with the *pelA* or *pelDE* mutants.

*BOS1* transcript accumulation was however lower after inoculation with the *prtE* and *pelDE* mutants as compared to that observed with the wild type parent 30 hpi. This might be related to the fact that the appearance of maceration symptoms are delayed after inoculation with these mutants as compared to wild type inoculation. However, the lack of *BOS1* activation with the *pelBC* mutant cannot be accounted for by differences in symptom severity since *pelBC* and *pelDE* mutants exhibited similar virulence on *Arabidopsis* (data not shown). These data indicate a major involvement of the PelB and/or PelC pectinases in the signalling leading to *BOS1* induction in the host cells.

### Different critical phases of infection progression are revealed during the *D. dadantii*/*bos1* mutant interaction

**Figure 2:**
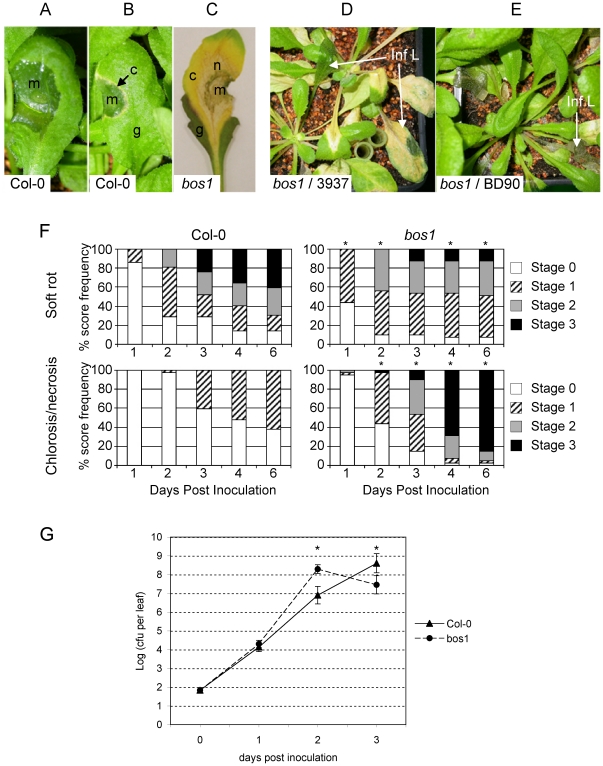
Disease development in the *Arabidopis bos1* mutant. Leaves were inoculated by needle wounding and depositing a 5 µl drop of a 10^4^ cfu/mL *D. dadantii* (strain 3937) suspension on Col-0 wild type and *bos1* mutant plants. **A:** typical maceration symptoms on Col-0 leaves. **B:** necrotic margin appearing around the maceration zone in Col-0 infected leaves. **C:** dried maceration zone surrounded by necrotic tissues in an almost totally chlorotic leaf of the *bos1* mutant. **D:** systemic necrosis on whole *bos1* plants 7 dpi. **E:** whole *bos1* plants inoculated with mycelium plugs of *B. cinerea* (strain BD90) 7 dpi. **m**: macerated tissue; **c**: chlorotic tissue; **g**: green tissue; n: necrosis; **Inf L**: infected leaf. **F:** kinetics of soft rot progression (top) and necrosis development (bottom) in Col-0 wild type plants (left) and *bos1* mutant (right). Inoculation of at least 40 Col-0 and *bos1* plants was performed on a single leaf per plant as previously described. Symptoms were scored during 6 days using 4 step scales as follows. Maceration scale: stage 0, no symptoms; stage 1, maceration around the bacterial suspension droplet; stage 2, spreading maceration; stage 3, maceration of the whole limb. Necrosis scale: stage 0, no necrosis; stage 1, necrosis surrounding the maceration zone (B); stage 2, necrosis of the whole infected leaf (C); stage 3, systemic necrosis (D). Asterisks indicate significant differences between Col-0 and *bos1* (Fisher test comparing the highest score at each day, p<0,05). **G:** *In planta* growth kinetics of *D. dadantii* on Col-0 wild type (dash line, triangles) and *bos1* mutant (dotted line, circles). Plants were inoculated as previously described. Each point corresponds to the average of at least 20 numerations and bars correspond to the standard errors. Asterisks indicate significant differences between Col-0 and *bos1* (Student's t-tests, p<0,01). The experiment has been performed three times with similar results.

The *bos1* mutant was previously described as hyper susceptible to the necrotrophic fungi *Botrytis cinerea* and *Alternaria brassissicola* but not to the biotrophic oomycete *Peronospora parasitica* nor to the bacterial pathogen *Pseudomonas syringae pv tomato* [33]. To investigate whether this mutant could be affected during *D. dadantii* infection, we analysed the disease symptoms produced on the *bos1* mutant after low bacterial inoculum deposition on wounded leaves. On Col-0 wild type plants, typical soft rot usually developed (Fig. 2A) spreading to the whole infected leaf, but in some cases, a chlorosis appeared around the maceration zone forming a yellow ring (Fig. 2B). In the *bos1* mutant such a chlorosis developed in almost all cases, expanded to the whole infected leaf (Fig. 2C) and spread systemically to the whole plant (Fig. 2D) leading to a complete death of *bos1* mutants. This chlorosis always turned into necrosis (Fig. 2C). The systemic necrosis of infected plants was not observed either after non-pathogenic enterobacterium inoculation (*E. coli*, strain 594, data not shown) or after *B. cinerea* mycelium (strain BD90) application (Fig. 2E). This indicated that a specific factor associated to *D. dadantii* might trigger this necrotic response.

The role that this necrotic response to *D. dadantii* inoculation might play in disease development has been analyzed by quantifying maceration symptoms, necrosis occurrence and bacterial growth in *bos1* and Col-0 plants. After inoculation with a 5 µl drop of a 10^4^ cfu/mL bacterial suspension, soft rot symptoms developed during the first 2 dpi in both genotypes but significantly faster in *bos1* leaves than in WT (P<0.05, Fig. 2F). Necrosis around the maceration zone developed from the second dpi in both genotypes but less frequently in Col-0 than in *bos1*. In wild type plants however, this necrosis always stayed restricted to the few plant cells surrounding the rotted tissue. In contrast, in *bos1* plants, the necrosis expanded to the whole infected leaves from 3 dpi onwards and affected rapidly the whole plant in most cases. This necrosis symptom was concomitant with an arrest of soft rot expansion and a drying of the maceration zone so that from the third dpi onwards, maceration symptoms did not evolve on the *bos1* mutant and the soft rot did not expand to more than one half of the infected leaves except in a few cases (Fig. 2F). In contrast, maceration continued to progress in WT leaves leading to the complete rotting of more than 40% of infected leaves at 6 dpi. To evaluate the consequence of necrosis on the infecting bacterial population, bacterial growth was monitored *in planta* in both genotypes (Fig. 2G). One day post inoculation, a 100-fold multiplication was observed in both genotypes. Two dpi, about 10-fold more bacteria were found in *bos1* infected leaves compared to wild type ones. On the contrary, a further increase in bacterial population was observed 3 dpi in wild type infected leaves while the number of bacteria declined in *bos1* leaves, correlating with the halt of maceration expansion and the appearance of the necrosis. In all chlorotic or necrotic systemic leaves tested, we never detected the presence of bacteria except for the rare leaves exhibiting maceration symptoms (data not shown). This implied that the local presence of bacteria is not required to spread this systemic necrosis, but rather that a diffusing factor might be emitted from the maceration site.

The differences observed in infection kinetics between Col-0 and *bos1* genotypes comparing both the maceration symptoms and *in planta* bacterial growth highlighted two critical phases of infection. During an early phase (until 2 dpi) the *bos1* mutant appeared significantly more susceptible compared to the wild type whereas, after 2 dpi, massive necrosis development in the *bos1* mutant around infected macerated sites was accompanied by a drop in bacterial population. This points to this process as an efficient defence mechanism developed by the host plant late during infection. Indeed this necrotic defence is exacerbated in the *bos1* mutant but also exists in wild type plants around rotten zones leading to maceration arrest.

### The *bos1* mutant is impaired in early ROS production after *D. dadantii* infection accompanied by less cell wall reinforcements

**Figure 3:**
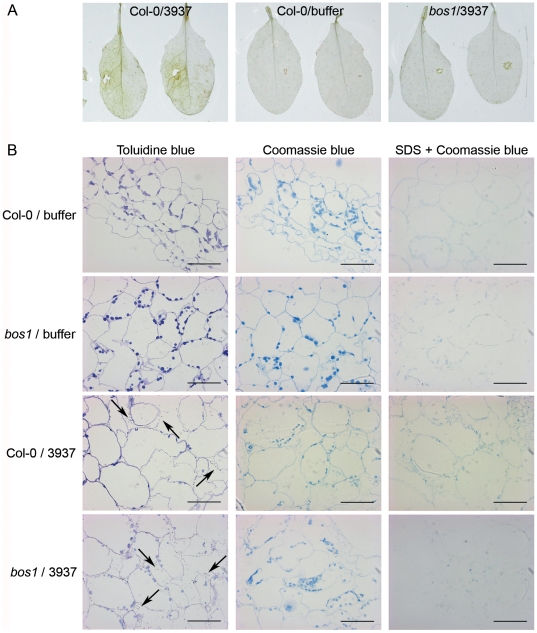
Early oxidative stress and protein cross-linking in Col-0 and *bos1* mutant leaves during *D. dadantii* infection. The leaves were inoculated by depositing about 50 bacteria or 5 µl phosphate buffer after needle wounding and staining was performed 1 dpi. **A:** oxidative stress analyzed by DAB staining of Col-0 wild type and *bos1* mutant infected leaves. **B:** analysis of protein cross-linking. Coomassie staining of leaf cells around the inoculated wound without SDS pre-treatment (center) or with SDS-removal of unbound proteins (right). Leaf cell structures and the presence of bacteria (indicated by arrows) were observed using toluidine blue stained sections (left). Plant genotype and inoculum are indicated on the left of the pictures. Bars represent 50 µm. All experiments have been performed at least three times.

Using a DAB staining that mainly revealed extracellular ROS production, an early NADPH oxidase-dependent oxidative stress, partly efficient as a defence reaction of *Arabidopsis* against *D. dadantii* infection, has been previously described [27]. To check the involvement of this ROS production in the *bos1* phenotype in the first stages of infection, we studied the oxidative stress induced 24 hpi in the *bos1* mutant using DAB staining. A much lesser DAB-detectable H_2_O_2_ production was observed in *bos1* than in WT leaves (Fig. 3A).

Extracellular oxidative stress could be involved in plant defence through its role in reinforcement of physical barriers limiting pathogen spreading. In particular, H_2_O_2_ production generates covalent links of cell wall proteins. Unbound proteins can be eluted during a SDS treatment of tissues whereas cross-linked proteins mainly remain included into the cell walls [37], [38]. *In situ* protein staining showed a comparable staining of cell walls before SDS treatment in Col-0 and *bos1* plants. However 24 hpi, after SDS treatment, no staining was observed in the area surrounding the maceration zone in *bos1* infected leaves, whereas a strong cell wall protein cross-linking was detectable in infected Col-0 leaves (Fig. 3B). These data are consistent with the H_2_O_2_ DAB staining.

### Systemic necrosis at late timepoints appears as an exacerbated defence mechanism in the *bos1* mutant

**Figure 4:**
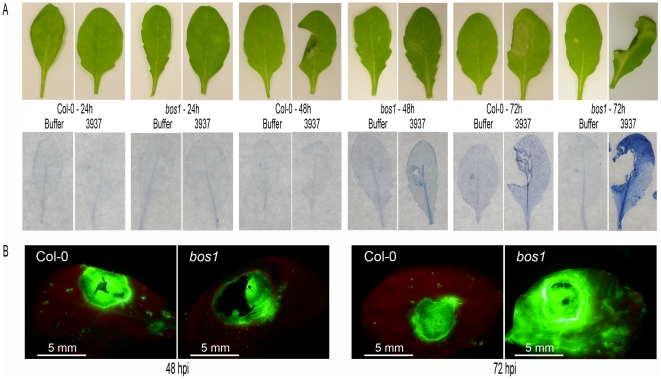
Enhancement of *D. dadantii* -induced cell death in *bos1* leaves. **A:** trypan blue staining of dead cells after 1–3 dpi. Maceration and necrosis symptoms were photographed (top) and the leaves were stained with trypan blue (bottom). One representative leaf of the eight stained in each case is presented. Col-0 and *bos1* leaves were inoculated, after needle wounding, by depositing 5 µl of buffer or 5 µl of a 10^4^ cfu/mL 3937 wild type bacterial strain suspension. **B:** intracellular oxidative stress 2 and 3 dpi analyzed by DCFH-DA staining of Col-0 wild type and *bos1* mutant leaves inoculated with *D. dadantii* after wounding. All experiments have been performed at least three times.

The spreading necrosis specifically observed in *bos1* after *D. dadantii* infection suggested the involvement of a cell death mechanism exacerbated in the mutant plants. We tested this hypothesis using trypan blue that stains dead cells. Staining was observed in both genotypes from 3 dpi onwards but it was clearly more intense and widespread in *bos1* leaves than in wild type ones (Fig. 4A). Interestingly, dead cells were already visible around the maceration zone of *bos1* infected leaves from 2 dpi, before any chlorosis - the first visible symptom of necrosis - was observed. This indicated that a cell death mechanism, strongly amplified in *bos1*, was induced in response to *D. dadantii* infection leading to a necrosis 3 dpi.

Since plant cell deaths are very often accompanied by extensive intracellular ROS production [39], H_2_O_2_ accumulation was monitored inside plant cells using DCFH-DA staining. No difference in intracellular H_2_O_2_ accumulation between *bos1* and the WT was observed with this method at early stages of infection. However, as it was previously observed during *B. cinerea* infection [33], we observed a much stronger oxidative stress in the *bos1* mutant than in the WT after *D. dadantii* infection 3 dpi when necrosis started (Fig. 4B). Intracellular H_2_O_2_ accumulation using DCFH-DA staining was still observed in the a*trbohD*-a*trbohF* double mutant line (data not shown) indicating that this late ROS production is NADPH oxidase-independent in our pathosystem.

## Discussion

Successful infections of compatible pathogens result from a subtle balance between the production of virulence factors and the host responses to pathogen invasion referred to as basal resistance [40]. In addition, pathogens like *D. dadantii* may colonize their host asymptomatically and intensive multiplication and maceration symptoms only occur when environmental conditions are favourable for disease expression [15]. The fate of symptom production might even be more complex as exemplified by the symptom appearance and progression differences in *Saintpaulia* - the plant from which the *D. dadantii* strain used in this study was isolated - and *Arabidopsis*. In *Saintpaulia*, there is a checkpoint in the symptom occurrence but, in most plants, once maceration is initiated, rotting proceeds to systemic maceration. In contrast, in *Arabidopsis*, maceration might stop at different stages during infection and up to 50–60% of the macerations stop prematurely within the inoculated leaf [11]. This arrest in maceration is usually accompanied by the necrosis of the plant cell layers directly adjacent to the macerated zone (see Fig. 2B). Analysis of the *Arabidopsis bos1* mutant revealed a tight control of this plant defence response to *D. dadantii* infection. The *bos1* phenotype associated to the *D. dadantii* infection is complex, highlighting two contrasting phases of the infection process in *Arabidopsis*. Indeed, maceration symptoms appeared and developed more rapidly in *bos1* as compared to wild type plants and, during the first two days post infection, *bos1* plants allowed up to a 10-fold higher bacterial multiplication. However, at later time points, a cell death process - as seen by a trypan blue staining - occurred around the macerated zone in most *bos1* plants leading to a necrosis. This necrosis then spread to the whole infected leaf and further systemically to the whole plant. This necrosis was accompanied by a maceration stop and a decrease in bacterial population in the infected area, indicating that this response is effective in stopping infection progression.

As observed during *B. cinerea* infection [33], *BOS1* transcripts accumulated in *D. dadantii* -infected plants from 12 hours post inoculation. This *bos1* activation was specifically dependent on the production and secretion of the PelB and/or PelC pectate lyases. *D. dadantii* secretes at least eleven pectinases via the type II Out secretion machinery, the five major ones being encoded by the *pelA* to *pelE* genes. These five genes are organized in two clusters in the bacterial chromosome, *pelADE* and *pelBC*. While inside a cluster, genes were highly related, proteins encoded by the two different clusters were more divergent [3], [9]. The 5 isoenzymes also differed in their enzymatic activities. While PelA, D and E showed an activity limited to substrates presenting a low degree of methylation, PelB and PelC were most active towards partially methylated pectin. The cleavage end products also varied, PelD and PelE producing mostly oligogalacturonic dimers while PelB and PelC generated predominantly trimers [35], [36]. Since both *pelBC* and *pelDE* mutants caused similar maceration rates on *Arabidopsis* leaves (data not shown), the loss of *BOS1* gene activation observed with the *pelBC* mutant did not appear to be correlated with differences in symptom production, but strongly pointed to its specific signal-dependent induction. It would be interesting to discriminate if such a signal results from the differences in amino acid sequence of the proteins - i.e. the proteins themselves being recognized - or from the various end products produced by the different isoenzymes. To that respect, it should be noted that activation of plant defence genes by pectin degradation products is well documented [41], [42]. Interestingly, Norman *et al.* [43], [44] showed that both PehA, the major *Pectobacterium* pectinase, and oligogalacturonides induce JA biosynthesis and related defence genes.

Extracellular ROS production generated by NADPH oxidases was reported as an early event in plant defence responses and ROS were proposed either to have direct toxic effects [45] or to act as signals that trigger induced defences to pathogen infection [29]. Accordingly, *Arabidopsis* responds to *D. dadantii* infection by the production of an oxidative burst mainly generated via the action of the AtrbohD NADPH oxidase. The absence of a functional *AtrbohD* gene in the host increased the susceptibility to *D. dadantii*, indicating the involvement of this enzyme in resistance to the bacterium [27]. Strikingly, no extracellular ROS production was observed at early time points in *bos1* infected leaves, pointing to a lack of the activation of the infection-induced oxidative stress in the *bos1* mutant. No differential expression of the *AtrbohD* gene has been observed during the first 30 hours after infection of *bos1* as compared to wild type plants (data not shown) indicating that the defect in the *bos1* mutant should not be at the *AtrbohD* transcriptional level. This lack of an early extracellular ROS production in *bos1* plants may account, at least partly, for the enhanced susceptibility to *D. dadantii* observed at the beginning of the infection. A direct anti-microbial effect of ROS on *D. dadantii* is unlikely since Miguel *et al.* [46] showed that there was no such effect for host-produced H_2_O_2_ in potato and tobacco. ROS production after pathogen attack was often accompanied by cell wall protein cross-linking, a reaction able to strengthen this physical barrier and to limit bacterial progression [45]. These two reactions were observed after *D. dadantii* infection of the resistant abscisic acid-deficient *sitiens* tomato mutant at the borders of bacteria-infiltrated areas, where bacterial containment was clearly visible [26]. Such protein cross-linking was clearly absent in *bos1* infected leaves and this lack might be one of the factors responsible for the more rapid bacterial spreading observed in the mutant. On the other hand, Torres *et al.* [28] showed that ROS production could suppress cell death in cells surrounding sites of NADPH oxidases activation. This cell death involved the salicylic acid signalisation pathway. Interestingly, as reported during infection with *Botrytis* [33], a strong accumulation of the PR1 salicylic acid-marker transcripts was observed in *bos1* leaves during *D. dadantii* infection (data not shown). We may therefore envision that the lack of early extracellular ROS production in *bos1* plants might be involved in the deregulation of necrosis spreading observed later during infection. Interestingly, the *bos1* necrotic phenotype looks like that observed with the lesion-mimic *lsd1* mutant impaired in a mechanism that protects *Arabidopsis* cells from death when confronted with oxidative stress signals [28], [47].

Plant cell death in response to pathogen attack has been often associated to ROS production [45]. Furthermore, increased ROS sensitivity has been proposed as a common factor in the various aspects of the *bos1* phenotype [33]. In *B. cinerea*-infected *Arabidopsis* plants, although ROS production could be detected early in the infection, significant ROS increase in *bos1* plants became apparent only as disease symptoms began to appear ∼2 days after inoculation. Similarly, in our pathosystem, intracellular ROS production visualized by DCFH-DA staining was detected in both *bos1* and wild type infected leaves 24 hpi with a similar intensity. However, when necrosis appeared, a strong accumulation of ROS that co-localized with necrosis was only observed in the *bos1* infected plants (Fig. 4B). Albeit responses of *bos1* plants to both *B. cinerea* and *D. dadantii* resulted in increased plant cell death and intracellular ROS production, the outcome of both infections varied drastically. *bos1* plants were highly susceptible to the fungus ([33]; Fig. 2) while *D. dadantii* -induced necrosis provoked an arrest of the maceration and of the bacterial multiplication thus appearing to be an efficient defence mechanism that is exacerbated in the *bos1* mutant. It should be noted that, on the contrary, no such effect on bacterial survival was observed after infection of *bos1* plants by both virulent and avirulent *Pseudomonas syringae* strains [33]. Interestingly, the efficiency of the barrier formed by dead tissues during *D. dadantii* infection, even when maceration started, could be related to the presence of bacteria colonizing intercellular spaces in asymptomatic regions ahead of the maceration zone ([27], our unpublished data). The *bos1* phenotype thus highlights the importance of this bacterial progression in healthy tissue for a successful infection and the inability of *D. dadantii* to survive in necrotized tissue.

The phenotype of the *bos1* mutant described in this work is consistent with the characterization of the mutant during its infection with *B. cinerea* previously published by T. Mengiste and co-workers [33]. Identification of the mutated gene has been strongly supported by the genetic link between the observed phenotype and the insertion of the T-DNA immediately 5′ to the ATG start codon of the open reading frame and by the restoration of the wild type phenotype of the *bos1* plants after introduction of a wild type copy of the *AtMYB108* gene [33]. A strong genetic link has also been observed in our hands between the *bos1* mutation and the necrotic phenotype during the interaction with *D. dadantii*. However, the stamen development phenotype of other alleles of *Atmyb108* described recently [48] as well as our search for other alleles conferring the *bos1* phenotype did not confirm the nature of the *bos1* mutation. As mentioned by Mandaokar and Browse [48], differences in phenotypes between the different alleles tested could be due to the unusual high accumulation of non-functional *BOS1* transcripts in the *bos1* mutant or to a complex molecular modification associated to the T-DNA insertion. This latter hypothesis could be supported by our failure to clone the right border of the inserted T-DNA.

Whatever the exact mechanism involved in the regulation, the data presented in this paper illustrate the complex interactions under play during *D. dadantii* infection, as revealed by the analysis of the *bos1* mutant. As previously reported [27], after *D. dadantii* infection, the plant builds up an early defence mechanism mediated by NADPH oxidases-dependent ROS production. We showed here that this might work at least partly via the ROS-dependent reinforcement of plant cell walls for bacterial containment. At later stages, when maceration occurs, the plant responds by inducing the death of the cells surrounding the infection site. This cell death leading to a necrosis appears to be an efficient mechanism against *D. dadantii* spreading and survival. The spreading of this necrosis whose induction is specific to *D. dadantii*, would however be deleterious for the plant; it is thus tightly regulated, a control lost in the *bos1* mutant. This control in turn would favour soft rot disease by acting as a potent inhibitor of this plant defence. The fate of *D. dadantii* infection appears to depend on the subtle balance between these effects.

## Materials and Methods

### Biological material and growth conditions

*Arabidopsis* seeds from the Col-0 ecotype were provided by I. Somssich (Köln, Germany) and the *bos1* mutant line [33] was kindly provided by T. Mengiste (West Lafayette, IN, USA). Plant cultures were performed under short day conditions at 24°C/19°C (8 h day/16 h night). The seeds were sown by batch in soil and grown for three weeks. Plantlets were then potted three plants per pot to allow growth for a further three weeks. The 6 week-old plants were incubated in small transparent containers with abundant watering to maintain 100% humidity 16 h before inoculation and throughout pathogenicity assays.

*D. dadantii*, *E. coli* and *Botrytis cirenea* strains used in this study are described in Table 1. Bacterial strains were grown at 30°C in Luria-Bertani LB medium and *B. cirenea* was grown on malt extract (CM) medium [49]. Media were solidified with 1.5% Difco agar. Liquid cultures were grown in a shaking incubator (220 rpm).

**Table 1:** Bacterial strains, fungus and primers used in this study.

strains	Description	Reference
*E. coli*		
594	Wild type	Lab collection
*E. chrysanthemi*		
3937	Wild type strain isolated from *Saintpaulia ionantha*	Lab collection
A3997	*prtE*::*uidA*-Km^R^	[11]
Ech132	*outC::uidA*-Km^R^	[54]
Ech457	*hrcC*::*uidA*-Km^R^	[11]
PMV4066	*pelA::*Ω	[55]
PMV4116	Δ*(pelADE)* Δ*(pelBC)*	[56]
PMV4120	*pelB::*Mu*d*IIPR13 *pelC::*Mu*d*II1734	[57]
PMV4072	*pelD::*Mu*d*II1734 *pelE::*Ω	Lab collection (M. Boccara)
*B. cirenea*		
Bd90	Isolated from grapevine	[58]

Primers for expression studies		
genes	Forward	Reverse
*TUB6*	TGGATCATGAGTGAGTGAAAAGA	ACCGACCAAACGAAAAGAAG
*BOS1*	GGGAAGAACGGACAACGAG	TGGCTATTCACATCACATTTCAA

### Quantitative Real-Time RT-PCR expression analysis

Total RNAs were purified as described in Lebeau *et al.* [11]. Briefly, 6 week-old *Arabidopsis* plants were infected by rapid immersion in a bacterial suspension (5.10^7^ cfu/ml) in 50 mM KPO_4_ pH 7 buffer containing 0.01% (vol/vol) of the Silwet L-77 surfactant (van Meeuwen Chemicals BV, Weesp, The Netherlands). Aerial plant tissues were collected at different time points post inoculation and ground in liquid N_2_ to a fine powder. RNAs were extracted in a guanidiumisothiocyanate extraction buffer and pelleted by centrifugation on a cesium chloride cushion. RNA samples were treated with RNAse-free DNAse I (Invitrogen) to get rid of any DNA contamination. First-strand cDNAs were then synthesized from 1–3 µg of total RNA using oligo(dT20) primer and M-MLV reverse transcriptase (Invitrogen), following the manufacturer's instructions. For quantitative Real-Time PCR analysis, cDNA was amplified using Maxima® SYBR Green/ROX qPCR Master Mix (Fermentas) according to manufacturer's license in an Applied Biosystems 7300 Real Time PCR System using the following conditions: 95°C for 10 min., 40 amplification cycles at 95°C for 15 s and 60°C for 60 s. Results were analyzed with Applied Biosystems Sequence Detection Software v1.3.1.

Primers are listed in Table 1. The *BETA-6 TUBULIN* gene (AT5G12250) was used as internal control to normalize the expression data for *BOS1*. The comparative quantitation method (ΔΔCt) was used to contrast the different conditions [50]. Ct values quantify the number of PCR cycles necessary to amplify a template to a chosen threshold concentration, ΔCt values quantify the difference in Ct values between a test (*BOS1*) and a control gene (*BETA-6 TUBULIN*) for a given sample, and ΔΔCt values are used for the comparison between two samples. ΔΔCt values were transformed to absolute values with 2^−ΔΔCt^ for obtaining relative *BOS1* transcript levels. References for relative transcript levels were set to 1. All assays were run in triplicate (biological replication) (Fig. 1A) or in duplicate (Fig. 1B) to control for overall variability.

#### Pathogenicity assays and bacterial numerations

Pathogenicity assays on 6 weeks-old *Arabidopsis* plants were performed as described in Lebeau *et al.* [11]. Bacteria were suspended in a 50 mM KPO_4_ pH 7 buffer to a concentration of 10^4^ bacteria/ml and inoculation was performed by wounding one leaf per plant with a needle and depositing a 5 µl droplet of this bacterial suspension (i.e. around 50 bacteria). About 40 plants were tested for each assay. Progression of symptoms was scored daily for 6 days. Assays were carried out at least in triplicate.

*B. cinerea* inoculation was performed as described in Soulié *et al.* [49]. Mycelia were grown on CM solid medium. Mycelium plugs were inverted onto the upper surface of one leaf per Arabidopsis plants. Inoculated plants were incubated under high humidity in the same conditions as described for bacterial inoculations.

For *in planta* bacterial numerations, about 120 *Arabidopsis* WT and *bos1* mutant leaves were infected as previously described. *In planta* bacterial growth kinetics were assessed during 3 days post-infection in short days conditions (8 h day/16 h night) with alternating temperatures (24°C/19°C). 20 to 30 inoculated leaves exhibiting maceration symptoms, except at 1 dpi where symptoms did not yet always appear, were ground in 200 µl KPO4 buffer, 50 mM, pH 7. The number of bacteria present in the resulting extract was determined by serial dilutions on LB plates. The bacterial numerations were performed in at least three independent experiments.

#### Histochemical assays

Detection of mainly extracellular H_2_O_2_ using 3,3′-diaminibenzidine (DAB, Sigma, St. Louis, USA) staining was performed as described by Torres *et al.* [51]. Twelve leaves from 12 different plants were inoculated as previously described in the pathogenicity assays and harvested for staining 24 h post inoculation i.e. when the first maceration symptoms appeared.

Detection method of mainly intracellular H_2_O_2_ using 2,7-dichlorofluorescein diacetate (DCFH-DA) was adapted from Zhang *et al.* [39] as described by Degrave *et al.* [52]. Inoculated leaves were harvested for staining each day during 3 days post inoculation. Leaves were immersed in a 300 µM DCFH-DA solution and vacuum-infiltrated. Green fluorescence was detected with an HQ510 1p emission filter. Experiments were performed with 12 leaves from 12 different plants in each experiment.

Cell death was studied using a trypan blue staining as described by Mauch-Mani and Slusarenko [53]. Inoculated leaves of Col-0 and *bos1* were harvested each day during 3 days post inoculation, photographed and immediately immerged into the staining solution prepared as follows: for 120 ml: 0,02 g of trypan blue (Acros, Organics) dissolved in a solution containing 10 ml phenol, 10 ml lactic acid, 10 ml glycerol, 10 ml water and 80 ml ethanol 96%. Samples were then incubated for 1 min in a boiling water bath and left 24 h at room temperature. Leaves were then distained three times with an aqueous chloral hydrate solution (2,5 g.ml^−1^, Sigma, Saint Louis, USA). Six leaves from different plants were analyzed for both phenotypes at each time point.

Protein cross-linking was studied as described by Thordal-Christensen [37] with the following modifications. Five leaves of Col-0 and *bos1* genotypes were inoculated and harvested 24 hpi. Tissues surrounding the inoculation site were fixed by 1 h incubation in a graded ethanol series (30, 50, 70, 90 and 100%), embedded in LR White resin (The London Resin Co., Hampshire, UK) and transverse semithin sections (1 µm thick) were cut with a diamond knife (Diatome histo, Bienne, Switzerland). Sections were then incubated during 2 min in toluidine blue (1%, Touzard & Matignon, Paris, dissolved in a 2,5% NaHCO_3_ aqueous solution) for structure visualization or in Coomassie Brilliant Blue (0,1%, Sigma, Saint Louis, USA, dissolved in a 40% ethanol, 10% acetic acid solution) during 15 min for protein staining. Prior to protein staining, cuts were incubated or not in 1% SDS at 80°C during 16 h to remove unbound proteins. About 10 cuts of each sample were used for each treatment. All sections were then observed with a Zeiss Axiophot light microscope (Zeiss, Oberkochen, Germany) and the presence of proteins observed as a blue staining.

All histological experiments were repeated at least three times giving consistent results.
